# Historical Tropical Forest Reliance amongst the Wanniyalaeto (Vedda) of Sri Lanka: an Isotopic Perspective

**DOI:** 10.1007/s10745-018-9997-7

**Published:** 2018-04-24

**Authors:** Patrick Roberts, Thomas H. Gillingwater, Marta Mirazon Lahr, Julia Lee-Thorp, Malcolm MacCallum, Michael Petraglia, Oshan Wedage, Uruwaruge Heenbanda, Uruwaruge Wainnya-laeto

**Affiliations:** 1 Max Planck Institute for the Science of Human History, Kahlaische Str. 10, 07745 Jena, Germany; 2 Research Laboratory for Archaeology and the History of Art, School of Archaeology, University of Oxford, Oxford, UK; 3 Anatomical Museum, College of Medicine and Veterinary Medicine, University of Edinburgh, Edinburgh, UK; 4 Leverhulme Centre for Human Evolutionary Studies, Department of Archaeology & Anthropology, University of Cambridge, Cambridge, UK; 5 Department of History and Archaeolpogy, University of Sri Jayewardenepura, Nugegoda, Sri Lanka; 6 Wariga Maha Gedara, Kotabakina, Dambana, Sri Lanka

**Keywords:** Tropical rainforest, Hunter-gatherers, Indigenous peoples, Stable light isotopes, Sri Lanka, The Wanniyalaeto

## Abstract

Headland and Bailey (1991) argued in *Human Ecology* that tropical forests could not support long-term human foraging in the absence of agriculture. Part of their thesis was based on the fact that supposedly isolated ‘forest’ foragers, such as the Wanniyalaeto (or Vedda) peoples of Sri Lanka, could be demonstrated to be enmeshed within historical trade networks and rely on crops as part of their overall subsistence. Yet, in the same volume and in the years that followed scholars have presented ethnographic and archaeological evidence, including from Sri Lanka, that counter this proposition, demonstrating the occupation and exploitation of tropical rainforest environments back to 38,000 years ago (ka) in this part of the world. However, archaeological and ethnohistorical research has yet to quantify the overall reliance of human foragers on tropical forest resources through time. Here, we report stable carbon and oxygen isotope data from historical Wanniyalaeto individuals from Sri Lanka, in full collaboration with the present-day members of this group, that suggest that while a number of individuals made use of agricultural resources in the recent past, others subsisted primarily on tropical forest resources as late as the 1800s.

**Electronic supplementary material:**

The online version of this article (10.1007/s10745-018-9997-7) contains supplementary material, which is available to authorized users.

## Introduction

During the 1970s and 1980s, tropical forests were seen as ‘pristine’ habitats, home to some of the last groups of hunter-gatherer societies untouched by agriculture and capitalism anywhere in the world (Stiles 1992). Countering this perception, Headland and Bailey (1991) argued that human forager habitation of tropical forest environments was virtually impossible without the consistent trade with agricultural societies noted in surveys of ethnographic and historical tropical forest societies. Key to their argument was the perceived dietary constraint imposed by highly spaced resources, seasonal flux, and the scarcity of energy-rich wild foods, such as fat-rich animals and carbohydrate-rich tubers (Hart and Hart 1986; Headland 1987; Bailey *et al.* 1989). Archaeologists quickly adopted these views. Since then, tropical forests have tended to be considered as ‘barriers’ to the movement of humans, from their first expansion beyond Africa during the Late Pleistocene onwards (Gamble 1993; Bird *et al.* 2005; Boivin *et al.* 2013).

As many have since pointed out, however, this does not mean that purely foraging lifestyles in tropical forests are impossible, now or in the past (Bahuchet *et al.* 1991; Balée 1994; Brosius 1991; Roberts and Petraglia 2015). In 1991 *Human Ecology* published a number of studies from different parts of the world that argued strongly against this thesis. Bahuchet *et al.* (1991) demonstrated that it was possible to gain sufficient carbohydrate resources from wild yams and other plant foods in the Central African rainforest, and that contemporary relationships between hunter-gatherers and farmers in this region distorted the perceived importance of such resources in the past. Similarly, Brosius (1991) discussed how the Penan hunter-gatherers of Sarawak, Borneo, manipulated the sago palm *Eugeissona utilis* to the extent that it could comfortably meet their calorific needs, while Dwyer and Minnegal (1991) and Stearman (1991) made comparable arguments for populations living in lowland Papua New Guinea and the Bolivian Amazon, respectively.

Importantly, archaeological research has definitively established that humans exploited tropical forest resources in the past. In the same volume, Endicott and Bellwood (1991) reviewed archaeological evidence at a series of Malaysian cave sites for the exploitation of tropical forest animals, including gibbons, flying foxes, and pigs, suggesting that foragers lived independently on these forests resources. Recent archaeological research in the rainforests of Southeast Asia and Melanesia has established the use of forests and forest edges by prehistoric hunter-gatherers since at least 45 ka (Barker *et al.* 2007; Summerhayes *et al.* 2010; Roberts and Petraglia 2015). South Asia, and particularly Sri Lanka, has also yielded an abundance of evidence for the hunting of semi-arboreal and arboreal mammals from 38 ka to 3 ka in tropical evergreen and semi-evergreen rainforest habitats (Wijeyapala 1997; Roberts *et al.* 2015a, 2017). Nevertheless, anthropological and archaeological studies have done little to test the overall contribution of wild tropical forest resources to human forager diets relative to other habitats or subsistence strategies.

In order to investigate this topic, we carried out stable carbon and oxygen isotope analysis of human tooth enamel from historical Wanniyalaeto (also known as ‘Vedda’) individuals from Sri Lanka. The status of these groups as dedicated foragers has been questioned since some of the earliest formulations of the hypothesis that tropical forests are not productive environments for human foragers. It was argued that by the 1800s, the Wanniyalaeto were part of trade networks in a globalized, colonial South Asia and made use of agricultural resources obtained through these networks (Bailey *et al.* 1989). However, numerous ethnographic accounts from the nineteenth and early twentieth centuries clearly indicate that despite the historical contacts, and indeed the long-standing presence of small-scale farming communities in Sri Lanka, these hunter-gatherers retained a mode of subsistence that relied significantly on forest resources (Seligmann and Seligmann 1911). Stable isotope analysis of human tooth enamel has the potential to directly test the use of forest resources and thus quantify the potential dietary and cultural effects of contact with farmers and imperial powers, and provide a historical dataset to compare to recent work on archaeological collections of forest foragers dating back to 36 ka (Krigbaum 2003, 2005; Roberts *et al.* 2015a, 2017).

## Forest Foraging amongst the Wanniyalaeto of Sri Lanka

Sri Lanka has been a key part of the broader debate regarding the suitability of tropical forests for long-term human forager subsistence. The Wanniyalaeto are a minority Indigenous group in Sri Lanka, whose language is commonly called “Vedda”, and are often linked to a pre-Sinhalese and Tamil period of occupation (Seligmann and Seligmann 1911). The term “Vedda” actually derives from the Tamil word for hunting, but has become a derogatory term in Sri Lankan society for anyone leading a rural, mobile way of life (Brow 1978; Boyle 2004). The Wanniyalaeto take pride in tropical forest foraging as a traditional way of life, and many historians and anthropologists (De Silva 1972, 1990; Bandaranayake 1985), as well as more recently geneticists (Ranaweera *et al.* 2014), have seen this as an isolating backdrop to cultural and genetic distinctiveness in this group relative to their neighbours. Between the eighteenth and the twentieth-first century, British colonialism, the growth of the Sri Lankan state education system, upheaval during the Sri Lankan civil war, and global capitalism have endangered this group’s survival, as well as their traditional culture and subsistence practices (Spittel 1961; Wickramasinghe 2016).

**Fig. 1:**
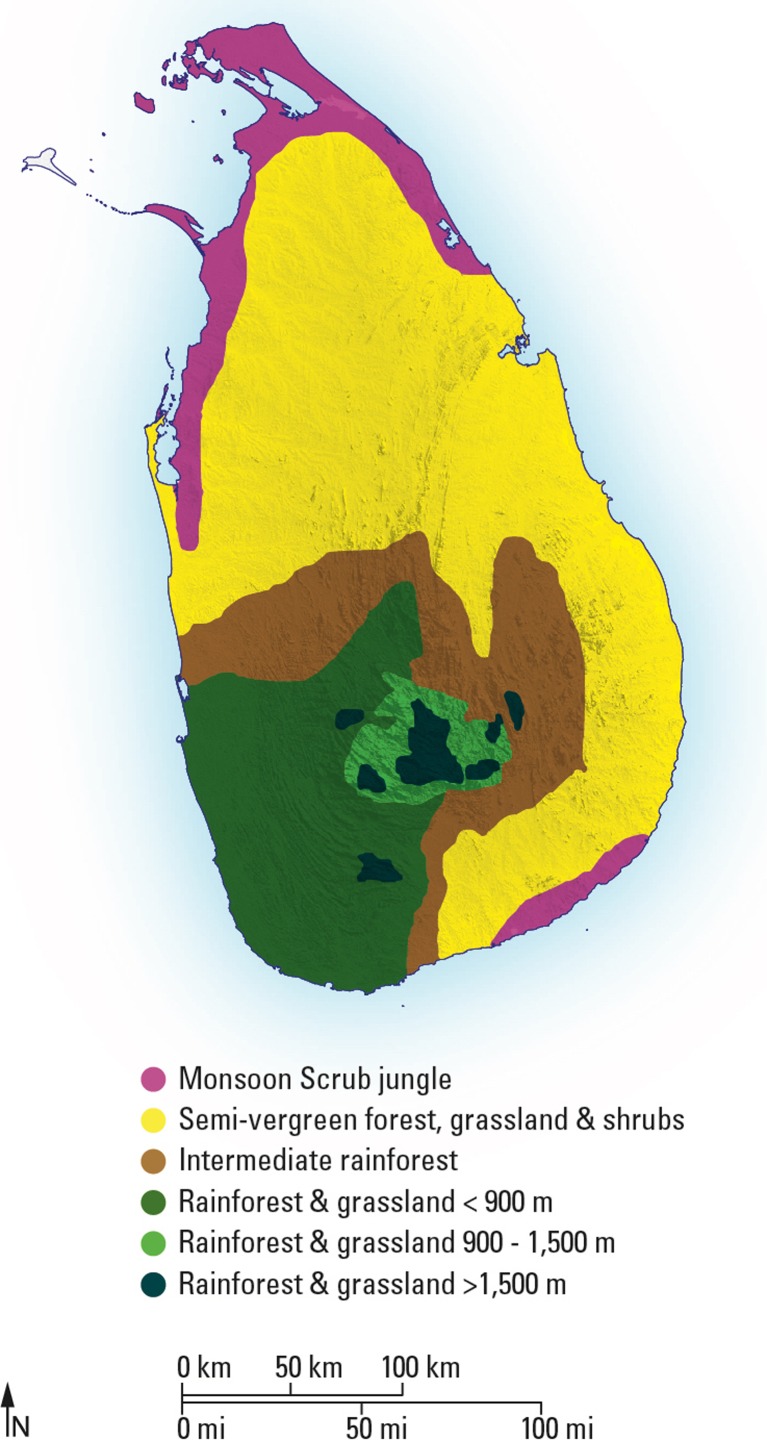
Map showing the vegetation zones of Sri Lanka after Erdelen (1988) and Roberts *et al.* (2015a)

The primary climatic parameter in Sri Lanka is precipitation. The highest annual precipitation within Sri Lanka occurs in a Wet Zone at the altitudinal gradient between the southwestern coastal plain and the central highlands (Roberts *et al.* 2015b), which receives between 4840 and 2201 mm of annual rainfall and is home to the island’s tropical flora of closed-canopy wet deciduous and tropical evergreen mixed dipterocarp forests (Ashton and Gunatilleke 1987; Gunatilleke *et al.* 2005) (Fig. 1). Tropical moist deciduous rainforest and tropical semi-evergreen forest extend into the Intermediate Zone of the island (Ashton and Gunatilleke 1987; Gunatilleke and Gunatilleke 1991), which forms an arc from the centre of its western coast to the southern tip, with mean annual rainfall of between 1701 and 2200 mm. The so-called ‘Dry Zone’ makes up the majority of Sri Lanka’s remaining landmass, with mean annual rainfall between 1001 and 1700 mm and is characterized by large expanses of shrubs and grasslands, with some ‘monsoon scrub jungle’ or ‘arid zone forest’ along the northern and southern coasts. Although Wanniyalaeto villages are today limited to the open ‘Intermediate’ rainforest and dry northern monsoonal jungles, during the nineteenth and twentieth centuries, particularly prior to British colonialism, they were widespread across the Wet and Intermediate rainforests of the island (Seligmann and Seligmann 1911; Knox 1981).

During the nineteenth and twentieth centuries Wanniyalaeto starch requirements were met by *Dioscorea* yams (Spittel 1924, 1961), wild date palms (*Phoenix pusilla*) and wild breadfruit (Sarasin and Sarasin 1893), and the seeds, stems, and rhizomes of various tropical forest plants (Spittel 1924, 1961). Honey was also reported as a major carbohydrate staple in the Wanniyalaeto diet (Seligmann and Seligmann 1911; Lewis 1915). Animal protein, including bee grubs, terrapins, tortoise, pangolin (*Manis crassicaudata*), bandicoot rats (*Bandicota bengalensis*), porcupine (*Hystrix indica*), giant squirrel (*Ratufa macroura*), hare (*Lepus nigricollis*), jungle fowl (*Gallus lafayetti*), mongoose (*Herpestes* sp.), and freshwater eels and fish, appears to have been the most important source of nutrition (Sarasin and Sarasin 1893). The Wanniyalaeto focused their subsistence in this regard on large-bodied monitor lizards (*Varanus bengalensis*), macaques (*Macaca sinica*), langurs (*Semnopithecus priam thersites*), pigs (*Sus* sp.), mouse-deer (*Moschiola meminna*), barking deer (*Muntiacus muntjak*), spotted deer (*Axis axis*), and sambhur (*Rusa unicolor*) (Bailey 1863; Sarasin and Sarasin 1908; Seligmann and Seligmann 1911), although their relative importance varied regionally. The basic method of procuring this larger game was bow and arrow, made entirely from available tree parts (Parker 1909; Seligmann and Seligmann 1911; Lewis 1915).

The Wanniyalaeto clearly developed specialized hunting and gathering strategies tuned to the capture of tropical forest prey and other products. Bailey *et al.* (1989), however, used historical evidence to argue that the Wanniyalaeto maintained economic contacts with agriculturalists as early as the seventeenth century, and that they did not completely rely on tropical forest foraging for their dietary requirements (Seligmann and Seligmann 1911; Knox 1981). A number of ethnographers recorded that the Wanniyalaeto traded forest produce, such as honey, wax, dried venison, and elephant tusks, with local Sinhalese communities for cultivars, such as rice and millet, alongside cloth, iron arrow-heads and axes, throughout the nineteenth and twentieth centuries (Sarasin and Sarasin 1893; Seligmann and Seligmann 1911; Spittel 1924; Morrison, 2014). Knox (1981) even noted that they served in the armies of Sinhalese kings. Seligmann and Seligmann (1911) also described ‘Coast Veddas’ in certain parts of the island. Nevertheless, while these Indigenous peoples undoubtedly exploited new connections, economic relationships, and resources, this does not necessarily mean that they were culturally and economically divorced from the forest and its dietary and other resources. Indeed, the limited forest use of the Wanniyalaeto today is primarily a result of land reorganization and the expansion of state systems, initiated under British rule and continued in the twentieth and twenty-first centuries (Spittel 1961). Furthermore, the recent diversification of the Wanniyalaeto economy does not mean that tropical forest resources were insufficient for subsistence without such trade, as documented from growing archaeological evidence in the region (Deraniyagala 1992; Perera *et al.* 2011; Roberts *et al.* 2017).

## Stable Isotope Analysis as a Direct Test of Human Forest Resource Reliance

The differing isotopes of elements such as carbon and oxygen respond differently to physical and biochemical processes because of their mass differences (Sharp 2006). This fractionation leads to different relative isotopic abundances in biological tissues that can be linked to environmental factors, such as temperature, or physiological factors, such as modes of photosynthesis. By convention the results are displayed in parts per thousand as the relative abundance of heavy (less abundant) to light (more abundant) isotope relative to an international standard (McKinney *et al.* 1950):

$$ \updelta\ \left({\mbox{\fontencoding{U}\fontfamily{wasy}\selectfont\char104}} \right)=\left({\mathrm{R}}_{\mathrm{sample}}/{\mathrm{R}}_{\mathrm{standard}}-1\right)\ast 1000, $$

where *R* is the ratio of the heavy to light isotope. Because the international standard is a marine limestone, which is relatively enriched in ^13^C and ^18^O, most of the δ-values for biological materials (such as plants, tooth enamel) are negative.

Differential fractionation during photosynthesis results in distinct non-overlapping δ^13^C values between C_3_ (−24 to −36‰ (global mean − 26.5‰)) and C_4_ (−9 to −17‰ (global mean − 12‰ (Smith and Epstein 1971)) plants. In a tropical context, this distinction is useful for studying the relative proportion of C_4_ grassland and C_3_ woodland or forest, or the relative proportion of C_4_ crops such as millet and C_3_ crops such as rice, in human diets and, indirectly, their associated environments (Krigbaum 2003, 2005; Roberts *et al.* 2015a). CAM plants may either fix atmospheric carbon in the manner of C_3_ plants or through a modified, diurnal C_4_ sequence that leads to δ^13^C values that are either within the range of C_3_ or C_4_ plants, or intermediate between the two (O’Leary 1981). However, while CAM plants can be found in tropical forests (Whitmore 1998), they are rare (Krigbaum 2001).

Within tropical forests, vegetation growing under a closed forest canopy is strongly depleted in ^13^C, due to low light (Farquhar *et al.* 1989) and large amounts of respired CO_2_ that remains semi-trapped under the canopy (van der Merwe and Medina 1991). As a result of the ‘canopy effect’, CO_2_, soils, and plants under a closed canopy have low δ^13^C values that are also reflected in the tissues of animals feeding in the same environments (van der Merwe and Medina 1991; Cerling *et al.* 2004). In the pre-fossil fuel era, tropical faunal tooth enamel with δ^13^C lower (i.e., more negative) than −14‰ suggests reliance on dense or closed canopy forest, while average values for herbivores in open landscapes would be about −12‰ and 0‰ for C_3_- and C_4_–feeders, respectively (Lee-Thorp *et al.* 1989a, b; Levin *et al.* 2008; Roberts *et al.* 2015a, 2017).

Stable oxygen isotope data from human tooth enamel can theoretically provide additional palaeoecological information about water resources and food. In tropical ecosystems, vegetation δ^18^O primarily reflects the source and nature of rainfall and then evaporative potential, which is dependent on relative humidity (Buchmann *et al.* 1997; Buchmann and Ehleringer 1998). The relationship between plant δ^18^O and evaporative potential can be used to infer levels of evapotranspiration and therefore, indirectly, canopy density (Roberts *et al.* 2017). For obligate drinking mammals such as humans, tooth enamel δ^18^O will reflect a combination of imbibed water, climatic and environmental effects on plants at the base of the foodchain, physiological factors of the individual and the species it consumes, and the diet of an individual.

Although bone collagen is typically the tissue of choice in human palaeodietary analysis because of the extra information about trophic level that can be obtained from stable nitrogen isotope analysis (Ambrose 1993), it is generally poorly preserved in tropical contexts (Krigbaum 2005). Tooth enamel is chosen here because it is more resistant to post-mortem degradation (Lee-Thorp *et al.* 1989b; Lee-Thorp 2008) and represents the ‘whole-diet’ for the period of enamel formation (Passey *et al.* 2005). This is between one to three years in most mammals depending on the tooth (Hillson 1996). Moreover, analysis of this tissue enables the data produced for historical foragers in Sri Lanka to be compared to a growing stable isotope dataset for human tooth enamel that has been accumulated for Late Pleistocene and Holocene Sri Lanka (Roberts *et al.* 2015a, 2017) and Holocene Southeast Asia (Krigbaum 2001, 2003, 2005).

## Methods

### Samples

We sampled teeth from groups labeled as ‘Vedda’ (*n* = 14) in the historical collections of the Duckworth Laboratory, University of Cambridge and the Department of Anatomy, University of Edinburgh (Table 1). All samples were donated to the museum in the late nineteenth or early twentieth centuries and are thought to belong to members of Wanniyalaeto culture (Table 1). In the process of repatriation negotiations involving these remains, the Wanniyalaeto elders indicated an interest in testing the forest reliance of their ancestors given the rapid disappearance of these subsistence sources from their diet in the twenty-first century as a result of relocation and the expansion of national education, urban, and agricultural infrastructure into their territories.

**Table 1:** Stable carbon and oxygen isotope ratios of historical Wanniyalaeto (“Vedda”) individuals analyzed in this study

Sample	Group	Accession number	Tooth	Source	Sex	δ^13^C (‰) (VPDB)	δ^18^O (‰) (VPDB)
VED1	“Vedda”	XXI.H.2	Lower left M3	Edinburgh	Male	−10.9	−4.8
VED2	“Vedda”	XXI.H.4	Lower left M3	Edinburgh	Male	−6.0	−5.1
VED3	“Vedda”	XXI.H.6	Upper left M2	Edinburgh	Male	−13.6	−5.8
VED4	“Vedda”	XXI.H.8	Upper right M2	Edinburgh	Male	−12.7	−2.9
VED5	“Vedda”	XXI.H.1	Lower right M3	Edinburgh	Male	−5.5	−4.2
VED6	“Vedda”	XXI.H.3	Lower left M3	Edinburgh	Male	−7.3	−7.3
VED7	“Vedda”	XXI.H.5	Upper left M1	Edinburgh	Male	−5.2	−5.4
VED8	“Vedda”	XXI.H.7	Upper left PM2	Edinburgh	Female	−14.1	−6.1
VED9	“Vedda”	XXI.G.18	Lower right M3	Edinburgh	Male	−14.2	−5.2
VED10	“Vedda”	AS.54.01	Lower left M2	Cambridge	Male	−9.3	−4.1
VED11	“Vedda”	6101	Lower left M2	Cambridge	Male	−10.0	−6.2
VED12	“Vedda”	6100	Lower left M2	Cambridge	Male	−6.8	−4.6
VED13	“Vedda”	1197	Upper right M1	Cambridge	-	−11.1	−4.3
VED14	“Vedda”	1196	Upper right M2	Cambridge	-	−12.0	−4.8

All samples come from historical collections at the Duckworth Laboratory, University of Cambridge, and the Department of Anatomy, University of Edinburgh

The Council of Wanniyalaeto Elders agreed to minimal sampling of tooth enamel for stable isotope analysis prior to repatriation. This project also forms a smaller part of larger collaborative research with the Wanniyalaeto that has been granted ethical approval from Friedrich Schiller Universität, Jena, Germany and the University of Jayawardenepura, Colombo, Sri Lanka. Close consultation with Indigenous peoples in this study has enabled a simultaneous scientific and cultural output and enriched interpretation of the results that will also be made available to the ‘Vedda Heritage Centre’ in Dambana, Sri Lanka, in a multi-lingual poster. When selecting the teeth to be sampled, teeth that grow late in the life of an individual were preferred so as to avoid any potential interference of weaning in the dietary signal. We focused on second or third molar teeth that form during the juvenile and early-adult periods of human life (Hillson 1996) (Table 1). Photographs were taken of all individuals prior to sampling and are available from the authors on request; these will, nevertheless, only be circulated with express permission from the Wanniyalaeto elders.

### Stable Isotope Analysis

The sampled teeth were cleaned using air-abrasion to remove any adhering external material. Enamel powder was obtained using gentle abrasion with a diamond-tipped drill along the full length of the buccal surface in order to maximize the period of formation represented by the resulting isotopic analysis for bulk samples. The resulting enamel powders were pre-treated using a protocol to remove organic and secondary carbonate contaminates. This involved a series of washes in 1.5% sodium hypochlorite for 60 min, followed by three rinses in purified H_2_O and centrifuging, before 0.1 M acetic acid was added for 10 min, followed by another three rinses in purified H_2_O (as per Lee-Thorp *et al.* 2012). Following reaction with 100% phosphoric acid, the evolved CO_2_ was analysed for stable carbon and oxygen isotopic composition using a Thermo Gas Bench 2 connected to a Thermo Delta V Advantage Mass Spectrometer at the Division of Archaeological, Geographic and Environmental Sciences, Bradford University. Carbon and oxygen isotope values were compared against two International Atomic Energy Agency (NBS 19, CO-8) standards and an in-house standard (MERCK). Replicate analyses of standards suggest that machine measurement error is *c.* ± 0.1‰ for δ^13^C and ± 0.2‰ for δ^18^O. Overall measurement precision was studied through the measurement of repeat extracts from a bovid tooth enamel standard (*n* = 20, ± 0.2‰ for δ^13^C and ± 0.4‰ for δ^18^O).

### Statistical Analysis

ANOVA tests were performed on human enamel δ^13^C and δ^18^O to determine if the “Vedda” populations differed from Late Pleistocene and Holocene tropical forest foragers from Sri Lankan archaeological sites in the Wet Zone of the island reported by Roberts *et al.* (2015a, 2017), as well as farmers and foragers from Late Holocene Borneo reported by Krigbaum (2003, 2005). A linear regression was also performed to test whether human enamel δ^13^C and δ^18^O were correlated for the ‘Vedda’ individuals sampled here. All statistical analyses were conducted using the free program R software (R Core Team 2013).

## Results

**Fig. 2:**
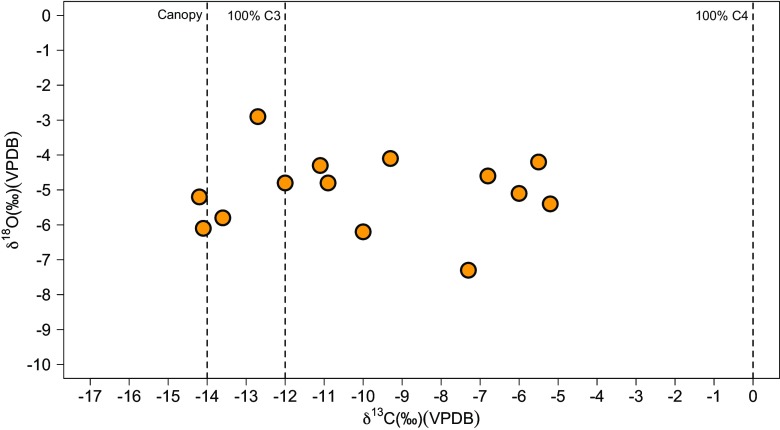
δ^13^C and δ^18^O measurements of Wanniyalaeto (“Vedda”) individuals analysed in this study. Dashed lines delineate estimated tooth enamel δ^13^C for individuals living under a dense “canopy”, individuals consuming 100% C_3_ resources, and individuals consuming 100% C_4_ resources from the literature (Lee-Thorp *et al.* 1989a, b; Levin *et al.* 2008; Roberts *et al.* 2015a, 2017)

We made no adjustment of the δ^13^C and δ^18^O data for human tooth enamel samples analysed for the Suess effect as δ^13^C_CO2_ in 1930 (after the origins of all of the dataset) differs from pre-industrial values by just *c.* 0.2‰ (Friedli *et al.* 1986) (Table 1; Fig. 2). The historical “Vedda” sampled have a wide δ^13^C range (−14.2 to −5.2‰), indicating varied individual reliance on closed-canopy C_3_, C_3_, and C_4_ or marine resources.

**Fig. 3:**
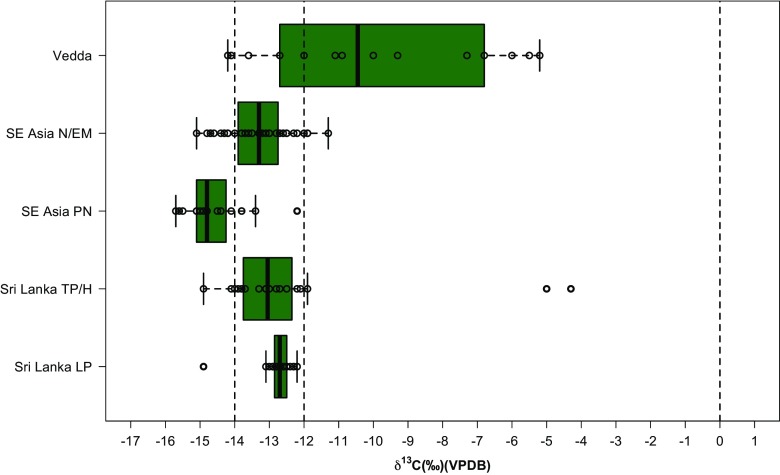
δ^13^C measurements of individuals from the Wanniyalaeto (“Vedda”) individuals analysed in this study as well as prehistoric human samples from Terminal Pleistocene/Holocene (Roberts *et al.* 2015a, 2017) (*c.* 12–3 ka) and Late Pleistocene (*c.* 36–13 ka) (Roberts *et al.* 2017) Sri Lanka, and Pre-Neolithic and Neolithic/Early Metal Age individuals from Sarawak, Borneo (Krigbaum 2003, 2005). The divisions for individuals living under a dense “canopy”, individuals consuming 100% C_3_ resources, and individuals consuming 100% C_4_ resources from the literature are again shown with dashed lines

The majority of individuals (~57%: 8 “Vedda”) have δ^13^C values between −15.0 and − 10.0‰, indicative of a clear dietary reliance on C_3_ resources. The two “Vedda” individuals with δ^13^C values between −15.0 and − 14.0‰ clearly document reliance on closed canopy forest resources (Fig. 2). “Vedda” individuals with values between −14.0 and − 10.0‰ could reflect more open tropical forest foraging, in settings akin to that of the Intermediate Zone rainforest today, or rice reliance, or varying proportions of the two. Over one third of the “Vedda” sample (*n* = 5) has δ^13^C values between −8.0 and − 5.0‰ (Fig. 3). While heavy reliance on marine foods could result in tooth enamel values as high as −10.0 and − 9.0 ‰, the higher values reported here clearly document some contribution of C_4_ resources to their diets.

The δ^18^O ranges of all groups are much smaller than those for δ^13^C (“Vedda” = −9.3 to −2.9‰) and the δ^18^O values obtained do not correlate with δ^13^C (Multiple R-squared = 0.01, *p*-value = 0.90, Adjusted R-squared = −0.08, p-value = 0.90)). This range is difficult to interpret given the potentially variable inputs of δ^18^O from food, different rainfall source, precipitation, and temperature in different parts of the island inhabited by these individuals. It does, however, fit comfortably within the range of variability documented for fossil human tooth enamel in the Wet and Intermediate Zones of Sri Lanka in the Terminal Pleistocene and Holocene (Supporting Information Tables S3-S4; >0.05) (Roberts *et al.* 2015a, b; 2017).

## Discussion

### Documenting Connections

The results of the stable carbon and oxygen isotope analysis of the South Asian Wanniyalaeto presented here confirm Bailey *et al.*’s (1989), Headland and Reid’s (1989), and Bailey and Headland’s (1991) arguments that many supposedly pristine ‘forest’ foragers were enmeshed in relationships with local agricultural communities or different environments by the nineteenth and twentieth centuries. At least five “Vedda” individuals document the contribution of C_4_ or open environment resources to their diets (Fig. 2). The higher δ^13^C values of these particular individuals could also be a result of hunting C_4_-feeding animals in the Dry Zone of Sri Lanka, although values higher than −9.0 ‰ are unlikely to result from the sole reliance on meat given constraints on the proportion of meat in the human diet. Similarly, while higher δ^13^C amongst some individuals could also be indicative of heavier reliance on marine protein, as noted in the ethnographic literature, heavy marine feeding is unlikely to account for values above −10.0 to −9.0 ‰. The contribution of marine foods could be tested in future by stable δ^13^C and δ^15^N analysis of bone collagen from similar historical populations (this was not done here to avoid more intensive damage to human remains being returned for repatriation).

As a result, the contribution of C_4_ resources to the diet is most likely to have been in the form of trade in C_4_ crops, such as millet, with local agricultural communities, as highlighted by Bailey *et al.* (1989). The dominance of this signal in certain individuals is interesting given a generally assumed dominance of rice agriculture among farmers in Sri Lanka from the Iron Age onwards (Deraniyagala 1992) (Fig. 2). The rising contribution of non-forest resources among the historical “Vedda” individuals analysed here is evident in statistical comparison of their δ^13^C with that of archaeological fossil samples (Supporting Information Tables S1-S2). The “Vedda” have significantly different δ^13^C relative to Late Pleistocene and Terminal Pleistocene/Holocene hunter-gatherers in Sri Lanka, as well as Pre-Neolithic hunter-gatherers and what have been interpreted as open-forest horticulturalists in Holocene Sarawak, Borneo (F(4,118) = 17.32, *p* < 0.05).

One of the first deviations from tropical rainforest reliance between 36,000–3000 years ago in the Wet Zone of Sri Lanka comes in the form of the incorporation of C_4_ resources, likely in the form of millet, into human diets during the Iron Age (*c.* 3 ka) (Roberts *et al.* 2015a, 2017). This can be seen in the two individuals within the Terminal Pleistocene/Holocene Sri Lankan group with values between −6.0 and − 4.0 ‰ (Fig. 3). These individuals are in contexts dated to 3 ka and are contemporaneous with continued rainforest foraging in Sri Lanka. Given that this crop is associated with dry, arid conditions, its appearance in the Wet Zone, as well as the historical Wanniyalaeto diets documented here, may be evidence for trade with agricultural populations in other environmental zones, or local significant clearance and changes in micro-climate (although the latter appears to be ruled out on the basis of faunal stable isotope analysis from 3 ka – Roberts *et al.* 2015a, 2017).

### Not ‘Pristine’ but Still Foraging in the Forest

Although the Wanniyalaeto demonstrate relationships with local agricultural populations, or the use of resources from non-forest environments, our data clearly show that this did not mean that forest foraging was no longer undertaken. Two individuals with δ^13^C values below −14.0‰ demonstrate a clear reliance on canopied tropical forest foraging. Individuals with δ^13^C values between −14.0 to −12.0‰ indicate reliance on more open C_3_ tropical forests or a combination of open and closed C_3_ environmental resources. As noted above, one of these C_3_ resources could be rice agriculture and δ^13^C values are similar to those early Iron Age Southeast Asian Neolithic and Early Metal age groups argued to be undertaking mixed horticulture and rice management in Sarawak, Borneo (Krigbaum 2003, 2005) (Fig. 3). Nevertheless, the Wanniyalaeto have been documented as *supplementing* their diet with rice, and so it seems more probable that those values sitting between −14.0 and − 12.0‰ reflect a considerable proportion of tropical forest foraging in more open rainforest conditions akin to the Intermediate rainforest today, with perhaps some contribution of rice.

**Fig. 4:**
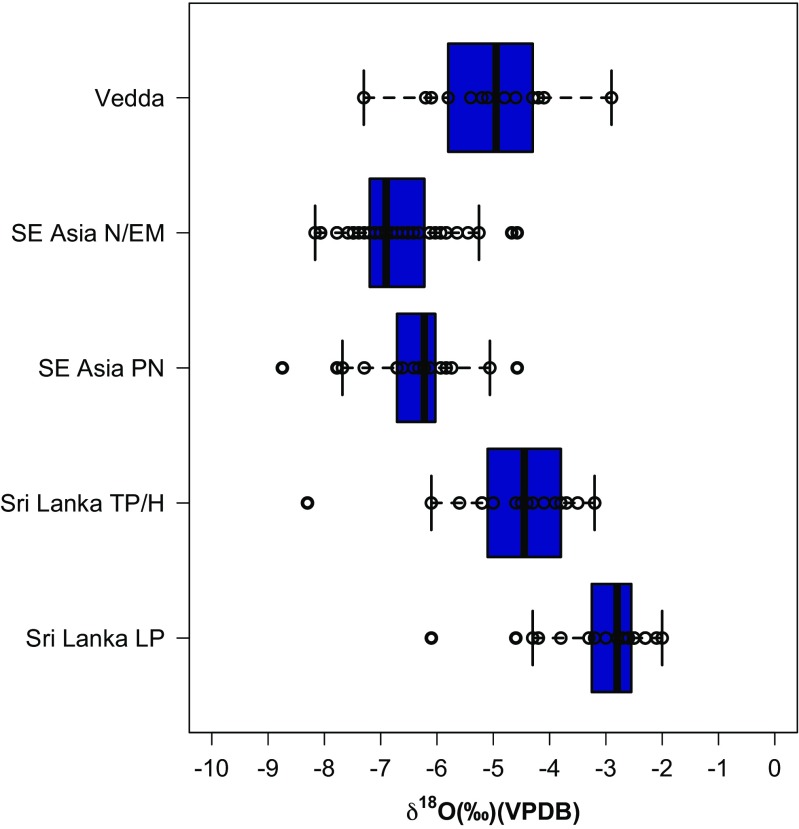
δ^18^O measurements of individuals from the Wanniyalaeto (“Vedda”) individuals analysed in this study as well as prehistoric human samples from Terminal Pleistocene/Holocene (Roberts *et al.* 2015a, 2017) (*c.* 12–3 ka) and Late Pleistocene (*c.* 36–13 ka) (Roberts *et al.* 2017) Sri Lanka, and Pre-Neolithic and Neolithic/Early Metal Age individuals from Sarawak, Borneo (Krigbaum 2003, 2005)

The ongoing dominance of tropical forest foraging among the “Vedda” samples analysed here may also be indicated by the δ^18^O of these individuals (Fig. 4). While the interpretation of these values in humans is difficult due to a number of sources of variation (including local climate, diet, and water sources), it is notable that the “Vedda” samples show no difference to Terminal Pleistocene and Holocene hunter-gatherers analysed from Sri Lanka, perhaps indicating that food and water sources (including alcohol brewing) from other groups played a relatively minimal role in overall water intake (F(4,118) = 63.88, *p* > 0.05) (Supporting Information Tables S3-S4). Furthermore, differences between “Vedda” and Holocene Southeast Asian Pre-Neolithic and Neolithic/Early Metal groups, as well as Late Pleistocene hunter-gatherers in Sri Lanka suggest that δ^18^O may prove to be useful in discerning geographical and climate-linked temporal changes in human water sources (F(4,118) = 63.88, *p* < 0.05). However, at present, this remains contentious.

Regardless, our results show that it was possible to obtain sufficient carbohydrate and protein from foraging wild resources in a tropical forest environment, in accordance with earlier anthropological observations and ecological assessments (Bahuchet *et al.* 1991; Brosius 1991; Dwyer and Minnegal 1991). While the consumption of millet, and perhaps rice, and therefore subsistence relationships beyond the forest are evident in some Wanniyalaeto individuals, there is no absolute transition. The Wanniyalaeto individuals included in the study may not be ‘pristine’ in the sense of exclusively securing dietary resources from tropical forests, but there is also a clear demonstration of agency in how different individuals engaged with new political and economic structures. These results are consistent with the large numbers of studies showing that the relationship between small-scale farming and foraging in the tropics is not clear-cut, and that foraging resources continued to be utilized well beyond the emergence of large-scale agricultural systems, often as a form of cultural resistance (e.g., Mercader *et al.* 2000; Krigbaum 2003; Kahlheber *et al.* 2009; Ferrier 2015).

Moreover, the demonstration of the possibility of forest reliance amongst the Wanniyalaeto aligns with growing evidence for the antiquity of reliance on tropical rainforest resources in Southeast Asia and South Asia (Sri Lanka). With the exception of two individuals from contexts dated to 3 ka mentioned above, all human individuals sampled from Late Pleistocene Sri Lanka, Terminal Pleistocene/Holocene Sri Lanka, and Pre-Neolithic Southeast Asia document specialized tropical forest subsistence, including an individual *c.* -15.0‰ in Late Pleistocene Sri Lanka that represents the earliest arrival of our species in South Asia *c.* 36 ka (Fig. 3) (Roberts *et al.* 2017). The tropical forests of South Asia and Southeast Asia at least have thus clearly provided an attractive environment for long-term foraging by *Homo sapiens,* and the role of forest resources extended long beyond the arrival of farming and colonialism in the region. Rainforest foraging is therefore a significant part of the cultural and ecological heritage of humans in Sri Lanka, and its ongoing importance to Wanniyalaeto Indigenous peoples should not be underestimated.

## Conclusion

Since the formulation of the hypothesis in the 1980s and early 1990s, the idea that tropical forests cannot support long-term human foraging in the absence of agriculture has been questioned from ecological, anthropological, and archaeological standpoints. Yet, a quantitative empirical assessment of the importance of tropical forest resources to ethnographic, historical, and prehistoric human foragers has remained elusive. Stable isotope analysis has emerged as one means of directly testing the degree of tropical forest resource reliance by a human individual during the period of enamel formation. The use of this method on historical ‘forest’ foragers in Sri Lanka has indicated that while some individuals did make use of other environments or resources, including those from trade with agricultural groups, this was not universally the case. This strongly indicates that long-term tropical forest foraging was not only possible, but that it remained a successful mode of subsistence for some Indigenous communities of Sri Lanka until the recent past. Furthermore, comparison of these data with a growing isotopic record from fossil humans in South Asia indicates that the tropical forests of this region have long been relied upon by our species for its subsistence. This conclusion not only urges caution with formulating definitive ecological assessments of human capabilities using modern or historical ethnography, but also highlights the importance of direct evaluations of human forest resource reliance, past and present.

## Electronic supplementary material

ESM 1 (DOCX 75 kb)
