# Supplementary material for: Historical Tropical Forest Reliance amongst the Wanniyalaeto (Vedda) of Sri Lanka: an Isotopic Perspective
Source: Hum Ecol Interdiscip J. 2018 Apr 24;46(3):435–44. doi: 10.1007/s10745-018-9997-7 (PMC6015624; doi:10.1007/s10745-018-9997-7)
Supplement: Supplementary file 1 — (DOCX 75 kb) [file 10745_2018_9997_MOESM1_ESM.docx]

Supporting Information for

**Historical tropical forest reliance in South Asia: an isotopic perspective**

Patrick Roberts^1,2*^, Michael Petraglia^1^, Marta Mirazon Lahr^3^, Malcolm MacCallum^4^, Thomas Gillingwater^4^, Julia Lee-Thorp^2^

^1^ Max Planck Institute for the Science of Human History, Jena, Germany

^2^ Research Laboratory for Archaeology and the History of Art, School of Archaeology, University of Oxford, Oxford, UK

^3^ Leverhulme Centre for Human Evolutionary Studies, Dept. of Archaeology & Anthropology, University of Cambridge, Cambridge, UK

^4^ Anatomical Museum, College of Medicine and Veterinary Medicine, University of Edinburgh, Edinburgh, UK

*correspondence to: Patrick Roberts

Max Planck Institute for the Science of Human History,

Kahlaische Str. 10,

07745 Jena,

Germany.

email: roberts@shh.mpg.de

**This PDF file includes:**

Supplementary Tables S1-S4

References

**Supplementary Table S1. Results of ANOVA for δ^13^C of “Vedda”, “Ceylon”, Sri Lanka Late Pleistocene, and Sri Lanka Terminal Pleistocene/Holocene individuals.**

|  | Degrees of freedom | Sum of squares | Mean square | F value | Pr(>F)* |
| --- | --- | --- | --- | --- | --- |
| Site | 4 | 195.7 | 48.92 | 17.32 | 0.000 |
| Residuals | 118 | 333.4 | 2.83 |  |  |

**Supplementary Table S2. Results of post-hoc Tukey pair-wise comparison of δ^13^C by ethnographic group. 95% confidence interval of difference is indicated alongside 95% probability of lower and upper bounds of this difference.**

| **Group** | **Difference** | **Lower** | **Upper** | **P-value*** |
| --- | --- | --- | --- | --- |
| SE Asia PN-SE Asia N/EM | -1.202 | -2.467 | 0.065 | 0.072 |
| Sri Lanka LP-SE Asia N/EM | 0.631 | -0.554 | 1.816 | 0.580 |
| Sri Lanka TP/H-SE Asia N/EM | 1.062 | -0.182 | 2.305 | 0.132 |
| Vedda-SE Asia N/EM | 3.455 | 2.037 | 4.872 | **0.000** |
| Sri Lanka LP-SE Asia PN | 1.833 | 0.389 | 3.276 | **0.006** |
| Sri Lanka TP/H-SE Asia PN | 2.263 | 0.771 | 3.755 | **0.001** |
| Vedda-SE Asia PN | 4.656 | 3.016 | 6.296 | **0.000** |
| Sri Lanka TP/H-Sri Lanka LP | 0.430 | -0.993 | 1.854 | 0.918 |
| Vedda-Sri Lanka LP | 2.823 | 1.245 | 4.402 | **0.000** |
| Vedda-Sri Lanka TP/H | 2.393 | 0.770 | 4.016 | **0.001** |

*p=<0.05

**Supplementary Table S3. Results of ANOVA for δ^18^O of “Vedda”, “Ceylon”, Sri Lanka Late Pleistocene, and Sri Lanka Terminal Pleistocene/Holocene individuals.**

|  | Degrees of freedom | Sum of squares | Mean square | F value | Pr(>F)* |
| --- | --- | --- | --- | --- | --- |
| Site | 4 | 235.9 | 58.97 | 63.88 | 0.000 |
| Residuals | 118 | 108.9 | 0.92 |  |  |

**Supplementary Table S4. Results of post-hoc Tukey pair-wise comparison of δ^18^O by ethnographic group. 95% confidence interval of difference is indicated alongside 95% probability of lower and upper bounds of this difference.**

| **Group** | **Difference** | **Lower** | **Upper** | **P-value*** |
| --- | --- | --- | --- | --- |
| SE Asia PN-SE Asia N/EM | 0.281 | -0.443 | 1.005 | 0.819 |
| Sri Lanka LP-SE Asia N/EM | 3.607 | 2.930 | 4.284 | **0.000** |
| Sri Lanka TP/H-SE Asia N/EM | 2.100 | 1.389 | 2.810 | **0.000** |
| Vedda-SE Asia N/EM | 1.663 | 0.852 | 2.473 | **0.000** |
| Sri Lanka LP-SE Asia PN | 3.326 | 2.501 | 4.151 | **0.000** |
| Sri Lanka TP/H-SE Asia PN | 1.819 | 0.966 | 2.672 | **0.000** |
| Vedda-SE Asia PN | 1.382 | 0.444 | 2.319 | **0.001** |
| Sri Lanka TP/H-Sri Lanka LP | -1.507 | -2.321 | -0.693 | **0.000** |
| Vedda-Sri Lanka LP | -1.944 | -2.846 | -1.042 | **0.000** |
| Vedda-Sri Lanka TP/H | -0.437 | -1.365 | 0.490 | 0.688 |

*p=<0.05
